# Protein molecular modeling techniques investigating novel *TAB2* variant R347X causing cardiomyopathy and congenital heart defects in multigenerational family

**DOI:** 10.1002/mgg3.401

**Published:** 2018-04-26

**Authors:** Thomas R. Caulfield, John E. Richter, Emily E. Brown, Ahmed N. Mohammad, Daniel P. Judge, Paldeep S. Atwal

**Affiliations:** ^1^ Department of Neuroscience Mayo Clinic Jacksonville FL USA; ^2^ Mayo Graduate School Neurobiology of Disease Mayo Clinic Jacksonville FL USA; ^3^ Department of Clinical Genomics Mayo Clinic Jacksonville FL USA; ^4^ Center for Inherited Heart Disease Johns Hopkins University Baltimore MD USA; ^5^ Division of Cardiology Medical University of South Carolina Charleston SC USA

**Keywords:** TGF‐beta activated kinase 1/MAP3K7 binding protein 2, whole‐exome sequencing

## Abstract

**Background:**

Haploinsufficiency of *TAB2* is known to cause congenital heart defects and cardiomyopathy due to its important roles in cardiovascular tissue, both during development and through adult life. We report a sibling pair displaying adult‐onset cardiomyopathy, hypermobility, and mild myopia. Our proband, a 39‐year‐old male, presents only with the above symptoms, while his 36‐year‐old sister was also notable for a ventricular septal defect in her infancy.

**Methods:**

Whole‐exome sequencing was utilized to identify the molecular basis of the phenotype found in two siblings. A molecular modeling technique that takes advantage of conformational sampling advances (Maxwell's demon molecular dynamics and Monte Carlo) were used to make a model of the mutant variant for comparative analytics to the wild‐type.

**Results:**

Exome sequencing revealed a novel, heterogeneous pathogenic variant in *TAB2*, c.1039 C>T (p.R347X), that was present in both individuals. This pathogenic variant removes just over half the residues from the TAB2 protein and severely impacts its functional ability, which we describe in detail.

**Conclusions:**

Analysis of the proband's family showed a history of cardiomyopathy, but no congenital heart defects or connective tissue disease. We highlight the heterogeneity in phenotype of *TAB2* pathogenic variants and confirm the pathogenicity of this new variant through neoteric protein modeling techniques.

## 1 INTRODUCTION

TGF‐beta activated kinase 1/MAP3K7 binding protein 2 (TAB2) is a protein encoded by *TAB2*, a gene located on chromosome 6q25.1 [OMIM #605101]. This protein serves a function in linking MAP3K7 and TNF receptor‐associated factor 6 (TRAF6) in the interleukin‐1 (IL1) signaling pathway, and is used in the activation of c‐Jun N‐terminal kinases (JNK) and nuclear factor kappa‐B (NFKB) (Takaesu et al., 2000). Research by Thienpont et al. (2010) has demonstrated that *TAB2* is also expressed in embryonic cardiac tissue in both humans and zebrafish. Knockdown of *TAB2* expression in zebrafish embryos caused delays in epiboly and convergent extension defects during gastrulation (around 12 hr postfertilization), ultimately resulting in significant heart failure around 36–48 hr postfertilization. Similarities in *TAB2* expression between humans and zebrafish at this stage of development, and the presence of 2 patients in an analyzed group with cardiac outflow tract defects and pathogenic missense variants in *TAB2*, prompted the researchers to conclude that haploinsufficiency of *TAB2* can cause congenital heart defects in humans (Thienpont et al., 2010). This conclusion is supported by additional reports detailing patients with pathogenic deletions or missense variants in *TAB2* leading to congenital heart defects, among other symptoms (Ritelli et al., 2018; Weiss, Applegate, Wang, & Batista, 2015).

In this report, we present two siblings having a novel, heterozygous nonsense pathogenic variant in *TAB2*, denoted c.1039 C>T (p.R347X). This variant creates a premature stop codon at residue 347 of the TAB2 protein and entirely removes its MAP3K7‐binding C‐terminal domain (Takaesu et al., 2000). The resulting *TAB2* variant likely interferes with proper IL1 signaling as a consequence, in addition to causing congenital heart defects in the siblings. We support this theory with novel protein molecular modeling techniques. Additionally, we discuss the patient's unique variant in *TAB2*, comparing his symptoms and family history to those of existing cases.

### 1.1 Case presentation

Our proband is a 39‐year‐old male who was first seen in our clinic at age 35. In his early medical history, he reports that he was hypermobile (Beighton score of 7/9), small in stature, and had a high‐arched palate. He has had several joint dislocations involving the digits and patellae, the first of which occurred around 12–14 years old. Due to family history including cardiomyopathy in three paternal uncles, father, and sister, the proband was investigated by a cardiologist at 16 years old. No heart problems were found, though his other symptoms were suggestive of an underlying connective tissue disorder and he was labeled as having “either Ehlers–Danlos or Loeys–Dietz syndrome.” Years later, the proband was found with a pulmonary artery aneurysm after he was hospitalized for a spontaneous pneumothorax in 2008. The proband was closely followed after this incident due to a worsening of the aneurysm and severe pulmonic regurgitation (PR).

At age 35, the proband's pulmonary artery aneurysm had grown to 5.7 cm. Aside from his severe PR, he was experiencing moderate mitral regurgitation, mild tricuspid regurgitation, severe left atrial enlargement, and biventricular failure with an ejection fraction of 22%. Surgery was now necessary. A pulmonary artery resection with pulmonary homograft valve 27 mm implantation was performed with resultant symptomatic improvement and an improved ejection fraction to 45%. The surgeons had noted myxoid degeneration in the pulmonary valve, supporting the presence of a connective tissue disorder. At age 37 he was found to have gallbladder stones and underwent a laparoscopic cholecystectomy. Days after the surgery, he returned to the hospital with acute respiratory distress, shortness of breath, and fever. Investigations revealed he had contracted Klebsiella pneumonia. He was hospitalized once again, and developed acute liver failure, acute renal failure, and respiratory failure requiring ventilator support, acute‐on‐chronic systolic heart failure, and pulmonary artery hypertension. The proband returned home after his multiorgan system failure had subsided, though he later experienced dyspnea and exercise intolerance. Pulmonary function tests were performed and found he had reduced left lung volume with restrictive disease of unknown etiology. This finding, along with his existing connective tissue disorder and pulmonary artery aneurysm, led to the proband being referred to medical genetics with suspicion of Loeys–Dietz syndrome.

During a genetics consultation, the proband agreed to multigene panels for thoracic aortic aneurysm and dissection and cardiomyopathy. The tests were negative, finding no pathogenic variants related to Loeys–Dietz, Ehlers–Danlos, or Marfan syndrome. As a next step, whole‐exome sequencing (WES) and mtDNA analysis were performed. WES uncovered the likely pathogenic variant c.1039 C>T (p.R347X) in *TAB2* in a heterozygous state, which we propose is responsible for the symptoms seen in the patient. This variant inserts a stop codon near the middle of the protein, resulting in TAB2 truncation and dysfunction.

Knowing of the likely pathogenic variant afflicting our proband, his sister was evaluated as well. She is a 36‐year‐old female who had a ventricular septal defect that was repaired in her infancy, right bundle branch and atrioventricular block, right ventricular dilation and diminished RV function, mild dilation of the aortic root, hypermobility (Beighton 5/9), unilateral sensorineural hearing loss, myopia, and mildly sloped shoulders. The similarity of her phenotype to the proband's was not a coincidence, as she was found to have the same *TAB2* variant. Probands mother was negative for the familial variant, suggesting likely paternal inheritance.

In the time since the proband's genetic testing, he has continued to follow with the cardiology clinic. EKG shows severe biventricular enlargement, an LV ejection fraction of 33%, a right bundle block, enlarged atria (the right more severely so), and mild aortic and mitral valve regurgitation. He developed brief episodes of chest pain, neck swelling with underlying lymphadenopathies, and volume overload. His pulmonary valve regurgitation and pulmonary artery aneurysm have worsened since his surgery at age 35, with the aneurysm reaching 5.2 cm. These symptoms prompted his placement on the cardiac transplantation list at age 39. The proband has been considered for a percutaneous pulmonary valve replacement or some form of mechanical circulatory support including a left ventricular assist device (LVAD), biventricular assist device (BIVAD), or total artificial heart while he waits for a transplant.

## 2 MATERIALS AND METHODS

### 2.1 Ethical compliance

Standard evidence‐based medical care was followed in treating the patient in this case and all procedures followed were in accordance with the ethical standards of the responsible committee on human experimentation (institutional and national) and with the Helsinki Declaration of 1975, as revised in 2000 (5). Written informed consent for genetic analysis and all other testing was obtained from the patient and his sister. Specific ethical approval was not sought for this case report.

### 2.2 Molecular modeling

The sequence of human TGF‐beta‐activated kinase 1 and MAP3K7‐binding protein 2 (known as TAB2), a protein encoded by *TAB2 gene,* was taken from the NCBI Reference Accession Sequence: NM_001292034: version NM_001292034.2, which is encoded for the amino acid sequence; and was used for computer assisted modeling. Monte Carlo simulations were performed on the mutant to allow local regional changes for full‐length 693 amino acids and when the p.R347X variant was introduced. We modeled this protein with the assumption that it could potentially be translated as a partially folded protein.

The X‐ray refinement for Monte Carlo was built using YASARA SSP/PSSM Method (Altschul et al., 1997; Hooft, Sander, Scharf, & Vriend, 1996; Hooft, Vriend, Sander, & Abola, 1996; King & Sternberg, 1996; Krieger et al., 2009; Qiu & Elber, 2006). The structure was relaxed to the YASARA/Amber force field using knowledge‐based potentials within YASARA. The side chains and rotamers were adjusted with knowledge‐based potentials, simulated annealing with explicit solvent, and small equilibration simulations using YASARA's refinement protocol (Laskowski, MacArthur, Moss, & Thornton, 1993). The entire full‐length structure was modeled, filling in any gaps or unresolved portions from the X‐ray. Refinement of the finalized models was completed using either Schrodinger's LC‐MOD Monte Carlo‐based (Altschul et al., 1997; Hooft et al., 1996, 1996; Krieger et al., 2009) (Videos [Link], [Link], [Link]).

Monte Carlo (MC) dynamics searching (MC search or Maxwell's demon molecular dynamics [MdMD]) was completed on each model for conformational sampling, using methods previously described in the literature (Caulfield, 2011; Caulfield & Devkota, 2012; Caulfield, Devkota, & Rollins, 2011; Caulfield & Medina‐Franco, 2011). Briefly, each TAB2 variant system was minimized with relaxed restraints using either Steepest Descent or Conjugate Gradient PR, then allowed to undergo the MC search criteria, as shown in the literature (Caulfield, 2011; Caulfield & Devkota, 2012; Caulfield & Medina‐Franco, 2011; Caulfield et al., 2011). The primary purpose of MC, in this scenario, is examining any conformational variability that may occur with different mutations in the region near to the mutation and possible effect on DNA binding or processing with TAB2.

## 3 RESULTS

### 3.1 MdMD, MC conformational searches, and free energy calculations

**Figure 1:**
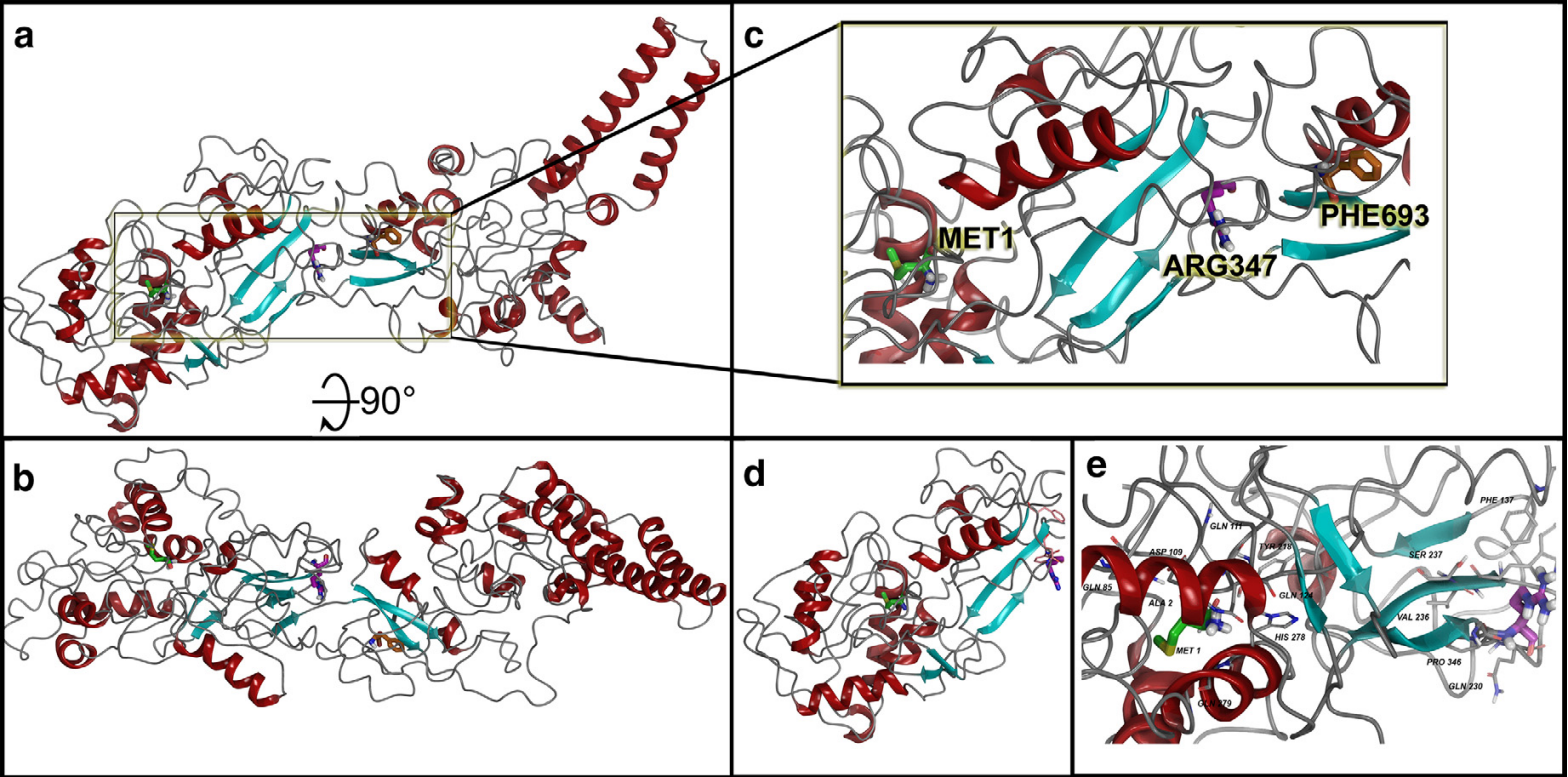
TAB2 molecular model for full‐length human sequence consisting of 693 amino acids and the truncation variant p.R347X. (a) Full‐length model for the entire TAB2 structure that shows two clear domains separated around amino acid 350–360, where R347 plays a role in interacting between the two domains. (b) Rotation of the full‐length model by 90° in *X*‐axis to better show the domains. (c) Zoom into the region around Arg347 and how the folded protein has Met1 and Phe693 folded within 35Å of each other. (d) Truncated TAB2 at R347X, showing smaller N‐terminus domain lobe. (e) Zoom into the N‐terminus domain lobe with residues within 12 Å of R347 shown and labeled. All protein ribbons are colored by secondary structure and residues shown in licorice rendering and using standard element coloring (C‐gray, O‐red, N‐blue, H‐white, S‐yellow) except for the highlighted residues (Met1‐green carbons, Arg347‐purple carbons, Phe693‐orange carbons)

Following the conformational sampling from MdMD and MC simulations for protein structure refolding, we calculated the free energy differences on a per residue basis and examined the effect on electrostatic surface. For WT versus the truncation variant p.R347X, we found the stability of the object from energetic calculations for ΔG per amino acid to remain relatively the same, such that WT and p.R347X were 0.954 and 0.951 kcal/aa*mol*Å^2^, respectively (Caulfield, 2011; Caulfield & Devkota, 2012; Caulfield & Medina‐Franco, 2011; Lopez‐Vallejo et al., 2011; Reumers et al., 2005; Schymkowitz et al., 2005; Zhang et al., 2013). This object stability did not indicate any changes in structure that were deleterious to function from immediate inspection, which the mutation foldx algorithm can provide. Thus we examined the local residues and determined an electrostatic calculation may be useful to explain the change in function. The molecular model for the full structure and its truncated form are given (Figure 1, Videos [Link], [Link], [Link]) using our established state‐of‐the‐art methods (Abdul‐Hay et al., 2013; Ando et al., 2017; Caulfield, 2011; Caulfield & Devkota, 2012; Caulfield, Fiesel, & Springer, 2015; Caulfield & Medina‐Franco, 2011; Caulfield et al., 2011, 2014; Fiesel et al., 2015, 2015; Lopez‐Vallejo et al., 2011; Puschmann et al., 2017; Zhang et al., 2013).

**Figure 2:**
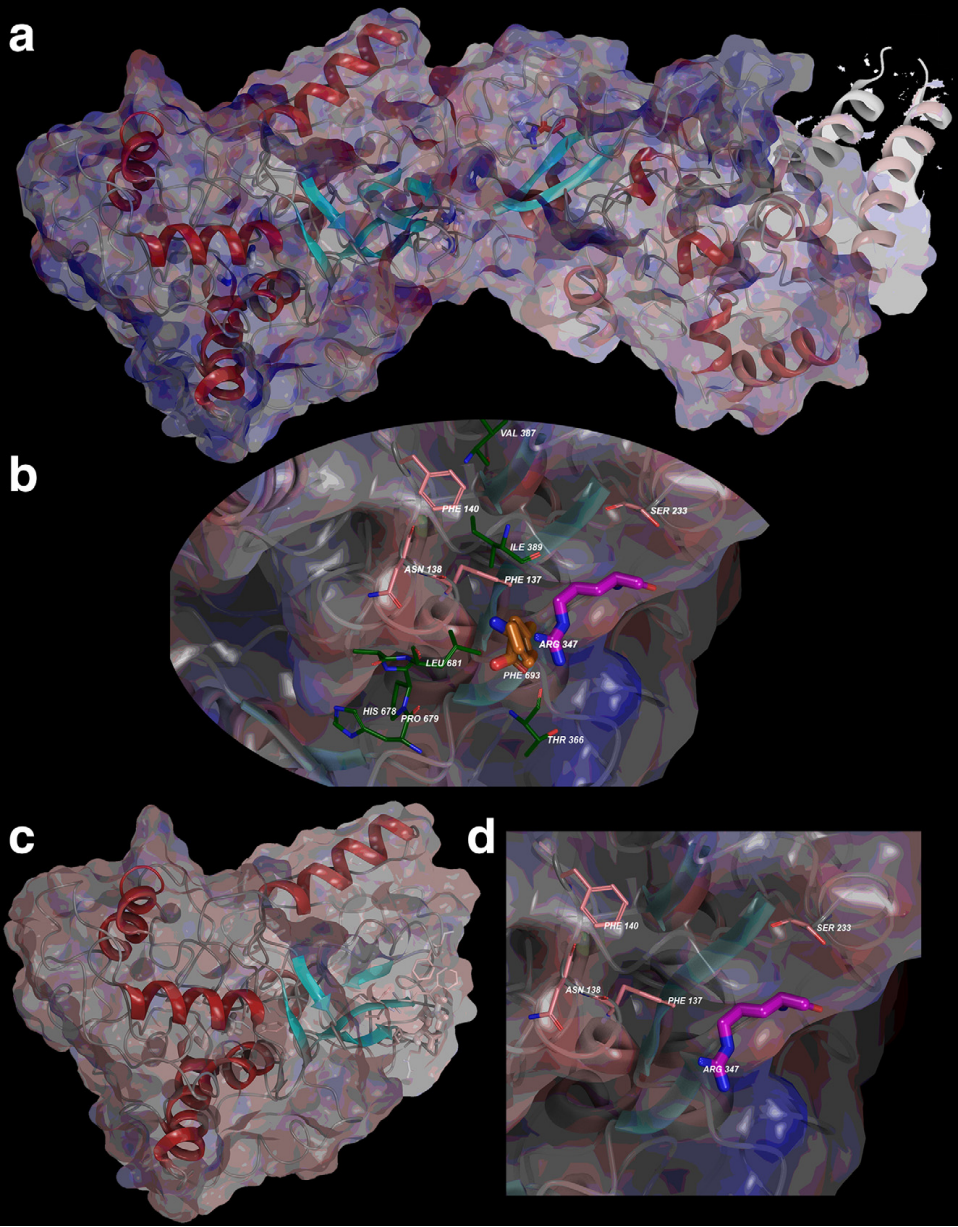
TAB2 electrostatic mapping for interaction potential. (a) Full‐length model for the entire TAB2 structure with electrostatics calculated from Poisson–Boltzman (PB) calculation overlaid onto structure. (b) Interacting residues between N‐terminus domain (1–358) and C‐terminus domain (359–693) are shown with electrostatics mapped onto. Amino acids Met1, Arg347, and Phe693 are colored as before—however, N‐term residues interacting with C‐term are colored with pink carbons and C‐term residues interacting with N‐term are colored with green carbons. (c) Deletion construct for the truncated p.R347X TAB2 model is given with electrostatics overlaid indicating increased negative charge. (d) Zoom into the region at the p.R347X site with only N‐terminus residues indicated

Local residues within the 12Å cutoff near the “cleavage” site include Phe137, Asn138, Phe140, Ile389, Thr366, Ser233, Val387, and Thre366, which tend to locate near the two domain halves found in the N‐terminus and C‐terminus lobes (Figures 1 and 2a,b). The N‐terminus lobe has four interesting amino acids involved in C‐terminus interactions in addition to the R347 residue, which includes Phe137, Asn138, Phe140, and Ser233. The residues from the C‐terminus side that are interacting with the N‐terminus lobe include residues Thr366, His678, Pro679, and Leu681 (Figure 2b).

Mapping electrostatics was accomplished using the Poisson–Boltzmann calculation for solvation on the entire 693 amino acid structure and the 347 amino acid structure. The effects of the changes were strongly pronounced on electrostatic distribution with a+3 KT/E cutoff for both. The WT particle (all 693 aa) has a distributed electrostatic charge that is both positive and negative and has zones between the two domains that hold together (N‐terminus interface is negative and C‐terminus interface is positive), which would only be perturbed by a competitive protein binder or substrate (Figure 2a). The p.R347X particle is predominately negative in charge distribution by comparison (Figure 2c). This could account for lowering its interaction potential with partner proteins in its pathway (Caulfield, 2011; Caulfield & Devkota, 2012; Caulfield & Medina‐Franco, 2011; Lopez‐Vallejo et al., 2011; Reumers et al., 2005; Schymkowitz et al., 2005; Zhang et al., 2013).

## 4 DISCUSSION

*TAB2* serves an important function both in the IL1 signaling pathway and in embryonic development of cardiac tissue (Takaesu et al., 2000; Thienpont et al., 2010). Pathogenic variants or haploinsufficiency of this gene leads to dysfunctional TAB2, which often has reduced MAP3K7 or TRAF6 binding ability (Weber, Wasiliew, & Kracht, 2010). Inability to bind these molecules results in lower expression of NFKB, which in turn favors cell necrosis over survival in cardiac tissue and often causes cardiomyopathy (Cheng et al., 2017). Deletions and pathogenic variants in *TAB2* can also cause haploinsufficiency in the embryonic stage of life, resulting in congenital heart defects (Thienpont et al., 2010). While it is true that not all individuals with deficiencies in TAB2 will experience congenital heart defects, many will experience cardiomyopathy or heart disease at some point in their lives (Cheng et al., 2017). Patients known to have a family history of congenital heart defects should be wary of this despite a potential lack of problems in childhood.

The pathogenic variant shared by our sibling pair, c.1039 C>T (p.R347X), has a variable phenotype which includes congenital heart defects and adult‐onset cardiomyopathy. As we noted before, the proband's sister had a ventricular septal defect as an infant which was surgically repaired. Conversely, the proband had no heart problems as a child and was informed by his cardiologist at 16 years old that he “likely would not need to return.” Despite this difference, both individuals ultimately developed similar symptoms of cardiomyopathy, including aortic/pulmonary arterial dilation, dilation of the ventricles, and conduction disease noted by a right branch bundle block. Additionally, these symptoms varied in severity between the two individuals. The proband has had persistent symptoms despite his homograft and is now awaiting heart transplant, while his sister has not required surgical correction since infancy, demonstrating inconsistent expression even within a sibling pair. The proband also displays more cardiac issues overall than his sister, who did not have valve regurgitation or myxoid degeneration of valves. Both siblings were notable for hypermobility, which has been well recognized in previous literature (Cheng et al., 2017; Ritelli et al., 2018). Both exhibited mild myopia, which has been recorded in one individual who coincidentally also has a pathogenic nonsense variant in *TAB2*, although this may well be multifactorial (Ritelli et al., 2018). The sloping shoulders seen in the sister and high‐arched palate seen in the proband seem to be unique features.

The molecular modeling from comparison of the wild‐type and variant indicates the natural stabilization induced by residues Phe137, Asn138, Phe140, Arg347 interacting directly with His678, Pro679, and Leu681, which are lost in variant p.R347X. Additionally, residue Thr366 interacts with both the N‐terminus and C‐terminus regions and we propose that any mutation of Thr366 (Ala, Gly, or Pro) would be useful screen for loss of function (lowered function) in TAB2 functional studies.

**Figure 3:**
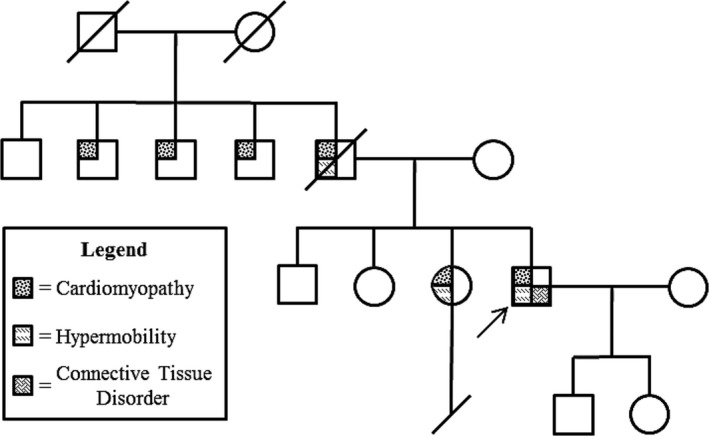
Family Pedigree. The proband's family pedigree demonstrating the multigenerational cardiomyopathy that can result from the p.R347X pathogenic *TAB2* variant

Through observation of the proband's extended family, we gain further support for the heterogeneous phenotype associated with his pathogenic variant of *TAB2* (Figure 3). Three of the proband's paternal uncles, his father, and his sister presented with cardiomyopathy. He shares his symptoms of hypermobility with his father and sister. During the proband's youth, the only characteristic sign of his TAB2 deficiency was his hypermobility, whereas his sister had a congenital heart defect that required surgical intervention. Despite this, he ultimately presented with the most severe phenotype of all family members having this pathogenic variant. This case therefore supports the conclusion that *TAB2* deficiency can present in various degrees of severity and at different stages of life, even within the same family. In summary, we report on a novel pathogenic variant in a large family leading to cardiomyopathy and other connective tissue features with variable expression, and confirm the pathogenicity by neoteric molecular modeling techniques.

## CONFLICT OF INTEREST

All authors declare no competing interests or disclosures.

## AUTHOR'S CONTRIBUTIONS

All authors above made substantial contributions to the conception or design of the work; or the acquisition, analysis, or interpretation of data for the work AND drafting of the work or revising it critically for important intellectual consent AND gave final approval of the version to be published AND agreed to be accountable for all aspects of the work in ensuring that questions related to the accuracy or integrity of any part of the work are appropriately investigated and resolved.
